# Effects of hydrogen-rich saline on early acute kidney injury in severely burned rats by suppressing oxidative stress induced apoptosis and inflammation

**DOI:** 10.1186/s12967-015-0548-3

**Published:** 2015-06-06

**Authors:** Song-Xue Guo, Quan Fang, Chuan-Gang You, Yun-Yun Jin, Xin-Gang Wang, Xin-Lei Hu, Chun-Mao Han

**Affiliations:** Department of Burn, Second Affiliated Hospital, School of Medicine, Zhejiang University, 88 Jiefang Road, Hangzhou, 310009 Zhejiang China; Department of Plastic Surgery, Binjiang Branch, Second Affiliated Hospital, School of Medicine, Zhejiang University, 1511 Jianghong Road, Hangzhou, 310000 Zhejiang China; Department of Orthopedic, Binjiang Branch, Second Affiliated Hospital, School of Medicine, Zhejiang University, 1511 Jianghong Road, Hangzhou, 31000 Zhejiang China

**Keywords:** Burn insults, Acute kidney injury, Hydrogen, Reactive oxygen species, Inflammation, Apoptosis

## Abstract

**Background:**

Early acute kidney injury (AKI) in severely burned patients predicts a high mortality that is multi-factorial. Hydrogen has been reported to alleviate organ injury via selective quenching of reactive oxygen species. This study investigated the potential protective effects of hydrogen against severe burn-induced early AKI in rats.

**Methods:**

Severe burn were induced via immersing the shaved back of rats into a 100°C bath for 15 s. Fifty-six Sprague–Dawley rats were randomly divided into Sham, Burn + saline, and Burn + hydrogen-rich saline (HS) groups, and renal function and the apoptotic index were measured. Kidney histopathology and immunofluorescence staining, quantitative real-time PCR, ELISA and western blotting were performed on the sera or renal tissues of burned rats to explore the underlying effects and mechanisms at varying time points post burn.

**Results:**

Renal function and tubular apoptosis were improved by HS treatment. In addition, the oxidation–reduction potential and malondialdehyde levels were markedly reduced with HS treatment, whereas endogenous antioxidant enzyme activities were significantly increased. HS also decreased the myeloperoxidase levels and influenced the release of inflammatory mediators in the sera and renal tissues of the burned rats. The regulatory effects of HS included the inhibition of p38, JNK, ERK and NF-κB activation, and an increase in Akt phosphorylation.

**Conclusion:**

Hydrogen can attenuate severe burn-induced early AKI; the mechanisms of protection include the inhibition of oxidative stress induced apoptosis and inflammation, which may be mediated by regulation of the MAPKs, Akt and NF-κB signalling pathways.

## Background

Acute kidney injury (AKI) is a devastating complication that affects patients exposed to severe burn injury [total body surface area (TBSA) ≥20%], which has been associated with a high mortality rate (from 50 to 100%) [1, 2]. The pathogenesis of AKI post burn is multifactorial and not completely understood [1, 2]. Early AKI may be attributed to intravascular hypovolemia, systemic vasoconstriction, early organ dysfunction or myoglobinuria, which appears during the first 5 days post burn and results from systemic inflammatory response syndrome (SIRS) or apoptosis; late AKI may be caused by sepsis, multi-organ failure or drug toxicity [2–5].

Combined with the apoptotic pathway, reactive oxygen species (ROS)-induced oxidative stress is involved in the development of renal dysfunction followed by AKI or other diseases [6, 7]. In addition, inflammation participates in the progression of renal impairment post burn, whereas ROS can induce inflammatory cytokine activities via the assistance of the nuclear factor (NF)-κB pathway [8, 9]. Mitogen-activated protein kinases (MAPKs), including p38 MAPK, c-Jun N-terminal kinase (JNK), and extracellular signal-regulated kinase (ERK), play important roles in the mediation of apoptosis, cellular proliferation and differentiation, and previous studies have verified that MAPKs are involved in the pathogenesis and protection against AKI caused by different stimuli [10]. ROS have been reported to be involved in MAPK activation [11, 12].

Ohsawa et al. first reported that molecular hydrogen (H_2_) is a novel, selective antioxidant that specifically neutralises the hydroxyl radical (·OH) and peroxynitrate anion (ONOO^−^), without disturbing metabolic oxidation–reduction or ROS-involved signals [13]. H_2_ is electrically neutral and much smaller than the oxygen molecule, it can easily penetrate cellular and intracellular membranes, which makes it highly effective in reducing cytotoxic radicals [14]. It also has a protective effect on oxidative stress-induced organ damage [9, 15, 16]. Furthermore, H_2_ has been shown to suppress inflammation and apoptosis in colitis, hepatitis, graft I/R injury and some chemical-induced organ injuries via the inhibition of inflammatory cell infiltration and the regulation of pro-inflammatory cytokine expression and inflammation/apoptosis-related signalling pathways [17–21]. Both the inhalation of H_2_ gas and the application of hydrogen-rich saline (HS) are effective routes for utilisation of the therapeutic properties of H_2_, as certified by prior experimental and clinical studies [15, 17, 22–25].

Given that ROS-based oxidative stress, and subsequent apoptosis/inflammatory response all play roles in the pathophysiological development and cytoprotection of burn-induced early AKI and that H_2_ has anti-oxidative stress, anti-apoptotic and anti-inflammatory effects, we hypothesised that H_2_ will have a protective effect on renal function after severe burn. In addition, details regarding the regulation of intrinsic signalling pathways were included in this study. Based on previous experiments, in this study, we selected HS as a H_2_ carrier and intraperitoneal (IP) administration as an effective and convenient application method [23].

## Methods

### Animals and treatment

**Figure 1:**
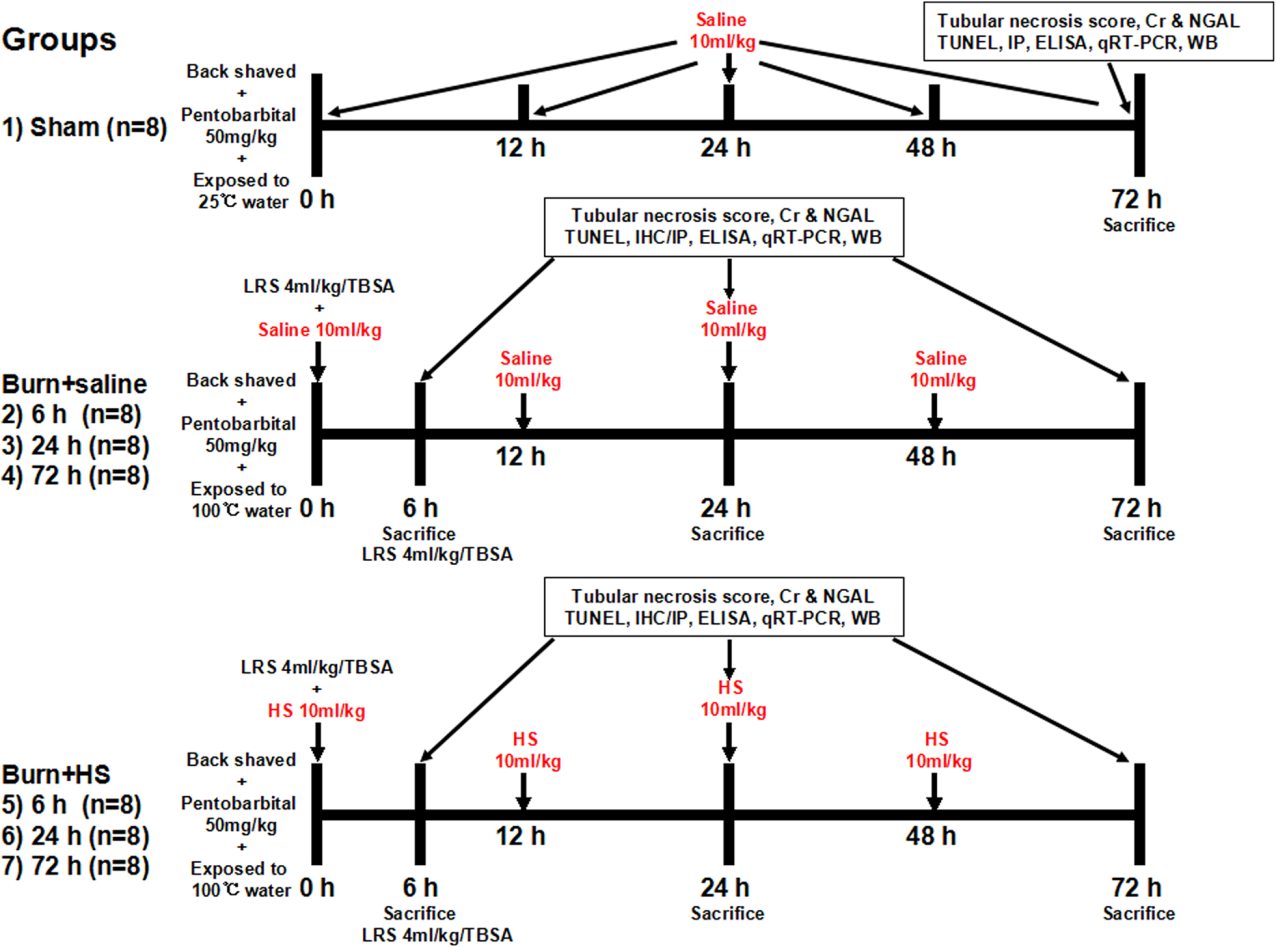
Experimental design and animal group classification. *Cr* creatinine, *HS* hydrogen-rich saline, *IHC* immunohistochemistry staining, *IF* immunofluorescence staining, *LRS* lactated Ringer’s solution, *NGAL* neutrophil gelatinase-associated lipocalin, *WB* Western blotting.

All experiments protocols on animals in this study were approved by the Committee on Animal Care of Second affiliated hospital, School of Medicine, Zhejiang University (No. 2015-140) and strictly abided by the National Institutes of Health Guidelines for the Care and Use of Laboratory Animals. Adult male Sprague–Dawley (SD) rats (weighing approximately 220–250 g) were purchased from the Animal Centre of Zhejiang Chinese Medical University (Hangzhou, China) and were housed on a 12-h light/dark cycle in an air-filtered unit with consistent temperature and humidity and free access to food and water. The animals were randomly assigned to seven groups (Figure 1), including the Sham group (saline, 10 ml/kg, immediate IP injection post water immersion) and three Burn + vehicle (saline, 10 ml/kg, immediate IP injection post burn) and three Burn + hydrogen saline (HS, 10 ml/kg, immediate IP injection post burn) groups (n = 8 per group). The rats in the Burn + vehicle groups and Burn + HS groups were sacrificed by overdoses of sodium pentobarbital at 6, 24 or 72 h post burn, while those in the Sham group were sacrificed at 72 h post water exposure. Both kidneys were dissected after cardiac perfusion with phosphate-buffered saline (PBS) (pH = 7.2) and were maintained in 10% formalin at 4°C or in a −80°C freezer for subsequent experiments.

### Severe burn model

After a SD rat was anaesthetised with sodium pentobarbital (50 mg/kg, IP injection), the shaved back of the rat was immersed into 100°C hot water for 15 s, generating a full-thickness dermal burn model with 40% TBSA [26]. The Sham group was exposed to 25°C water after anaesthesia [26]. Liquid resuscitation with lactated Ringer solution (LRS) at 4 ml/kg/TBSA was performed via IP injection immediately and 6 h after the operation. All rats were housed in individual cages and given 0.25 mg/kg buprenorphine by subcutaneous injection immediately and every 12 h post burn for analgesia. The pain and distress scale, reported previously, were conducted immediately and every 6 h after recovering from anaesthesia to evaluate the pain condition of rat models and instruct pain-reliving therapy [27].

### HS preparation

Hydrogen-rich saline was prepared as previously described [16, 23, 28]. In general, H_2_ was dissolved in 0.9% saline for 6 h under 0.4 MPa pressure to a supersaturated condition using HS-producing apparatuses from the Department of Diving Medicine, the Second Military Medical University, Shanghai, China. Gas chromatography (Biogas Analyzer Systems-1000, Mitleben, Japan) was applied to monitor the hydrogen concentration (maintained at greater than 0.6 mmol/l in HS [13]).

### Drug administration

The rats in the three-time-point post burn (6, 24, 72 h) Burn + HS groups received HS (10 ml/kg) by IP injection immediately after SAH induction, which was re-administered every 12 h before sacrifice. An equal volume of 0.9% saline (10 ml/kg) was IP-injected into the SAH models of the three Burn + vehicle groups (6, 24, 72 h) immediately and every 12 h after SAH induction. The Sham group was given 0.9% saline immediately and every 12 h post warm-water exposure.

### Histological evaluation

The fixed kidneys were cut into 7-μm-thick sections for haematoxylin and eosin (HE) staining, and the tissue slices were observed under the microscope. Histological changes were scored based on the percentage of renal cortical tubules that expressed epithelial necrosis, and these changes were ranked as 0: normal, 1: less than 10%, 2: 11–25%, 3: 26–75%, and 4: greater than 75%. Ten high-magnification files for every slice were randomly selected for blinded observation.

### Renal function evaluation

Rat blood samples were collected to measure the serum levels of creatinine (Cr) via a clinical chemistry analyser system and kits (Prochem-V, Drew Scientific, Dallas, TX, USA). The serum neutrophil gelatinase-associated lipocalin (NGAL) levels were detected in the various groups using a Rat NGAL ELISA kit (Boster, Wuhan, China) according to the manufacturer’s instructions.

### Measurement of redox potential, lipid peroxidation and antioxidant enzymatic activity

The oxidation–reduction potential (redox potential, ORP) value was determined using the HI3131B electrode (Hanna Co, Ltd, Italy) according to the provided instructions. For detection, 0.5% renal tissue homogenate was injected into the device under airtight conditions at 25.2°C. The renal tissue homogenate reacted with a thiobarbituric acid reactive species (TBARS) assay kit (KeyGEN, Nanjing, China), and this reaction was used to obtain the malondialdehyde (MDA) levels. Tissue superoxide dismutase (SOD), glutathione peroxidase (GSH-Px) and catalase (CAT) activities were measured using commercial assay kits from KeyGEN Biotech (Nanjing, China) according to the manufacturer’s protocols. The absorbance values were measured using a microplate reader (Model 680 Microplate Reader, BIO-RAD, CA, USA).

### TUNEL staining for apoptosis

The commercial cell death detection kit was purchased from Roche Diagnostics (Indianapolis, IN, USA). The stained slices were observed and photographed under a microscope (DM5500B, Leica, Solms, Germany), and the apoptotic index was determined as the percentage of apoptotic cells versus the total number of cells counted in a blinded manner.

### Immunohistochemistry (IHC) staining

Paraffin-embedded tissues (5-μm-thick slices) were examined by IHC and IF staining. Some sections were incubated with anti-myeloperoxidase (MPO) antibodies (Abcam, Cambridge, UK) overnight at 4°C. Then, they were incubated with goat anti-rabbit secondary antibody (Boster, Wuhan, China), and visualised with a 3,3-diaminobenzidine (DAB) kit (Boster, Wuhan, China). Finally, the mounted sections were observed and photographed under a microscope at 200× magnification (DM2500, Leica, Solms, Germany).

### Detection of renal tissue MPO activity

Tissue homogenate were obtained for the detection of MPO activity using a rat-specific ELISA kit according to the manufacturer’s instructions (Lianshuo, Shanghai, China).

### Quantitative real-time PCR (qRT-PCR) analysis of renal tissue

The expression levels of TNF-α, IL-1β, IL-6, IL-10 and ICAM-1 were analysed via qRT-PCR. Briefly, total RNA was isolated from tissues with TRIzol Reagent (Invitrogen, Carlsbad, CA, USA) and RNase-Free DNase I (Qiagen, Duesseldorf, Germany). The SuperScript First-Strand Synthesis System for reverse transcription PCR (RT-PCR) (Invitrogen, Carlsbad, CA, USA) was applied to synthesise cDNAs, and RNA and cDNA concentrations and purities were measured via BIO-RAD spectrophotometry (SmartSpecTM Plus, BIO-RAD, CA, USA). The primers (Table 1) were designed using Primer Premier 6.0 software and were synthesised by Shanghai Biological Engineering Co., Ltd. (Shanghai, China). PCR amplifications were conducted using the Power SYBR^®^ Master Mix (Invitrogen, Carlsbad, CA, USA) in an iQ™ 5 Real-time PCR system (BIO-RAD, CA, USA). Expression levels were assessed relative to that of 18S rRNA, as an internal standard, and the details are shown in Table 1. Relative quantification of the target gene expression levels was conducted using the 2^−∆∆Ct^ method.

**Table 1:** The oligonucleotide primers used for PCR amplification

Gene	Genbank accession	Primer sequences (5′–3′)	Size (bp)	Annealing (°C)
Rat TNF-α	NM_012675.3	GCCACCACGCTCTTCTGTCTACTG TGGGCTACGGGCTTGTCACTC	152	64
Rat IL-1β	NM_031512.2	GCTTTCGACAGTGAGGAGAATGAC CTGCTGTGAGATTTGAAGCTGGAT	126	64
Rat IL-6	NM_012589.2	TGACAGCCACTGCCTTCCCTAC CAATCAGAATTGCCATTGCACAA	169	64
Rat IL-10	NM_012854.2	GCACTGCTATGTTGCCTGCTCTT GAGCATGTGGGTCTGGCTGACT	111	64
Rat ICAM-1	NM_012967.1	CACAAACGACGCTTCTTTTGCTCT CCCCTCTTGCCAGGTCCAGTT	144	64
Rat 18S (reference substance)	M11188	GAATTCCCAGTAAGTGCGGGTCATA	105	64

### Western blotting analysis

The protein samples of the right kidneys were mixed with loading buffer and subjected to SDS-PAGE. The transferred membranes were subsequently blocked and incubated overnight at 4°C with the following primary antibodies: anti-cleaved caspase-3, anti-Akt, anti-p-Akt, anti-p38, anti-p-p38, anti-ERK, anti-p-JNK, anti-JNK (all from Santa Cruz, CA, USA), anti-NF-κB p65 (both from Abcam, Cambridge, UK), and anti-p-ERK (Cell Signalling Technology, Boston, USA). β-actin (Santa Cruz, CA, USA) was blotted on the same membranes and served as the control. The protein bands were detected with SuperSignal® West Dura Extended Duration Substrate (Pierce, USA) and X-ray Film (Kodak, USA) and were then analysed with Bandscan 5.0 software and compared with β-actin.

### Statistical analysis

The data are presented as the mean ± standard error of the means (SEMs). GraphPad Prism version 5.01 (San Diego, CA, USA) and SPSS 19 (SPSS, Chicago, IL, USA) were used for the statistical analyses. The comparisons between the Sham group and each of the burn groups, which were treated with saline or HS, were conducted using Mann–Whitney U tests. Multiple comparisons among groups treated with saline or HS at different times were analysed with two-way analysis of variance (ANOVA) followed by Bonferroni’s post hoc tests. A value of p < 0.05 was accepted as statistically significant.

## Results

### HS attenuates the severity of renal tubular damage and the elevation of serum Cr and NGAL levels in severely burned rats

**Figure 2:**
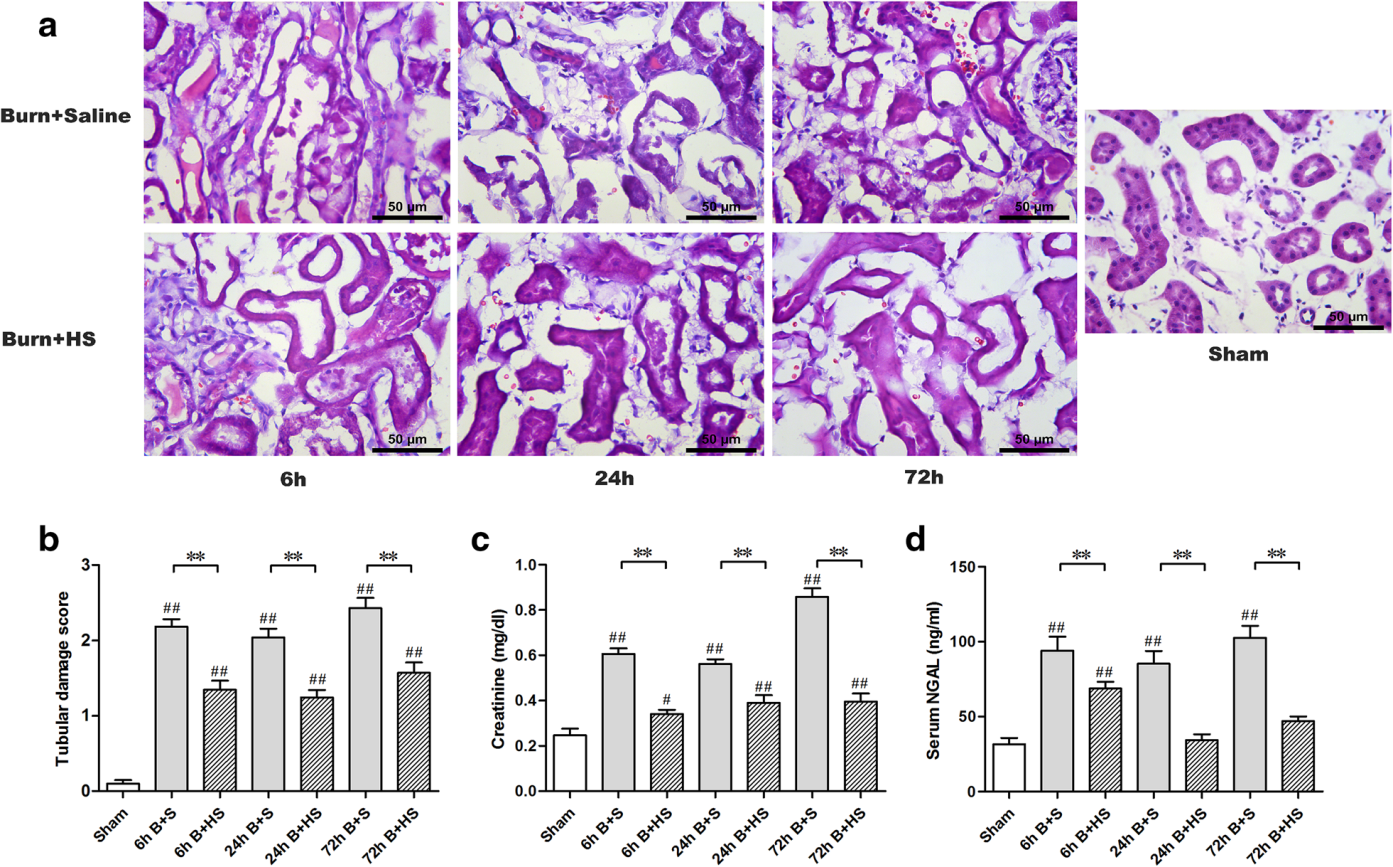
Histological and laboratory evaluations of renal function in the early stage of post burn. Representative HE-staining images of different paired groups (saline or HS treatment) manifested histological evidence of renal tubular damage at different time points post burn at a magnification of ×400. Several injurious changes appeared on the renal tubules after burn insult (**a** *upper row*); HS treatment resulted in an attenuation of these effects (**a** *lower row*). Furthermore, the tubular damage scores provided quantitative verification (**b**). With respect to the blood examination, serum creatinine (**c**) and NGAL (**d**) levels showed similar remarkable elevations after burn, which indicated the development of burn-induced early renal dysfunction. The sample size was n = 8 for each group. The results were expressed as the means ± SEMs. **p < 0.01, vs Burn + saline; ^#^p < 0.05, ^##^p < 0.01, vs Sham.

HE-stained slices were analysed via histological examination (Figure 2a). The tubular damage scores significantly increased in the rats at 6 h post burn and remained elevated at 72 h post burn, although a slight decrease was observed at 24 h (all p < 0.05, vs Sham) (Figure 2b). Similarly, significant elevations in serum Cr levels were observed post burn at three time points (all p < 0.05, vs Sham) (Figure 2c). For HS treatment, the tubular damage scores in the rats were significantly decreased at the different time points post burn injury (all p < 0.05, vs corresponding Burn + saline) (Figure 2b); in addition, the Cr levels were reduced (Figure 2c). After severe burn insult, the serum NGAL levels in the rats were markedly elevated at 6 h and were improved at 24 h but remained significantly higher than those of the Sham group (all p < 0.05) (Figure 2d). The NGAL levels clearly increased again at 72 h post burn (p < 0.05) (Figure 2d). HS treatment caused a remarkable decline in the NGAL levels in the sera of burned rats at various time points after burn (all p < 0.05), and we observed the lowest NGAL level of the three Burn + HS groups in the 24-h group (Figure 2d).

### HS relieves severe burn-induced oxidative stress in the renal tissues of rats

**Figure 3:**
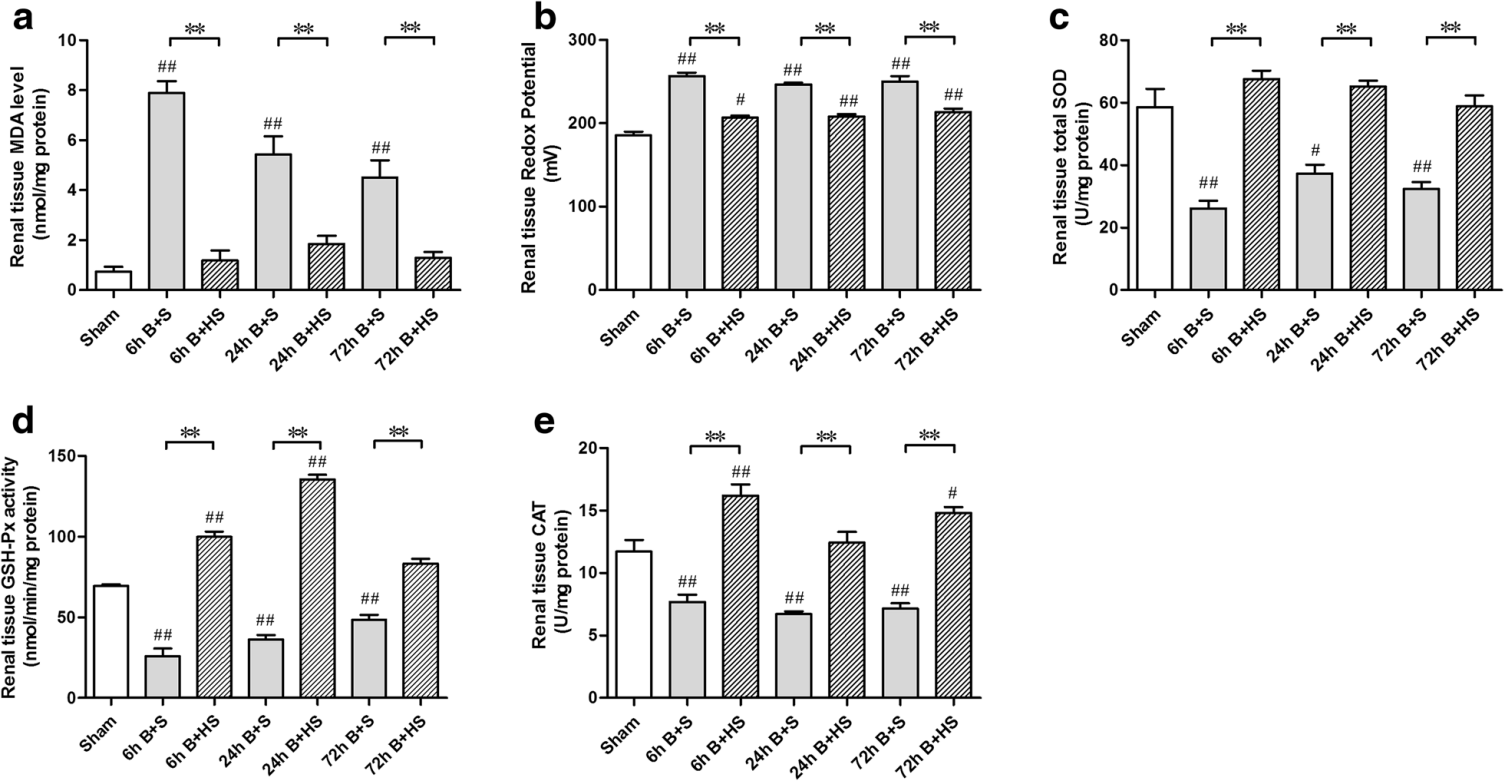
Assessment of oxidative stress and antioxidant enzyme activities in the kidneys of burned rats and the effects of HS treatment. After burn injury, the renal tissues of the rats displayed significant elevations in the ORP value in the different groups compared with the Sham group (**b**). Additionally, the MDA levels in the rat renal tissues presented remarkable augmentations after burn (**a**), with simultaneous declines in the activities of endogenous antioxidant enzymes (SOD, GSH-Px, and CAT) (**c**–**d**). Following the application of HS for different lengths of time post burn, significant reductions were observed in the ORP and MDA levels, and antioxidant enzyme activities increased (**a**–**e**). The sample size was n = 8 for each group. The results were expressed as the means ± SEMs. *p < 0.05, **p < 0.01, vs Burn + saline; ^#^p < 0.05, ^##^p < 0.01, vs Sham.

After burn injury, all three time point groups displayed significant elevations in their ORP values in renal tissues compared with the Sham group (all p < 0.05, vs Sham) (Figure 3a). HS treatment gave rise to marked reductions in the ORP value in the corresponding groups (all p < 0.05, vs corresponding Burn + saline) (Figure 3a). Additionally, severe burn induced a sharp increase in the MDA level at 6 h (p < 0.05, vs Sham) (Figure 3b). Subsequently, the extent of MDA elevation gradually decreased at 24 and 72 h, despite the levels of both groups being obviously higher than that of the Sham group (both p < 0.05, vs Sham) (Figure 3b). HS treatment significantly decreased the increases in MDA levels at all three time points (all p < 0.05, vs corresponding Burn + saline) (Figure 3b). Extensive burn injury also reduced the activities of endogenous antioxidant enzymes (total SOD, GSH-Px, CAT) (all p < 0.05, vs Sham) (Figure 3c–e), and HS application significantly reversed the reduction of antioxidant enzyme activities post burn (all p < 0.05, vs corresponding Burn + saline) (Figure 3c–e).

### Apoptosis evaluation

**Figure 4:**
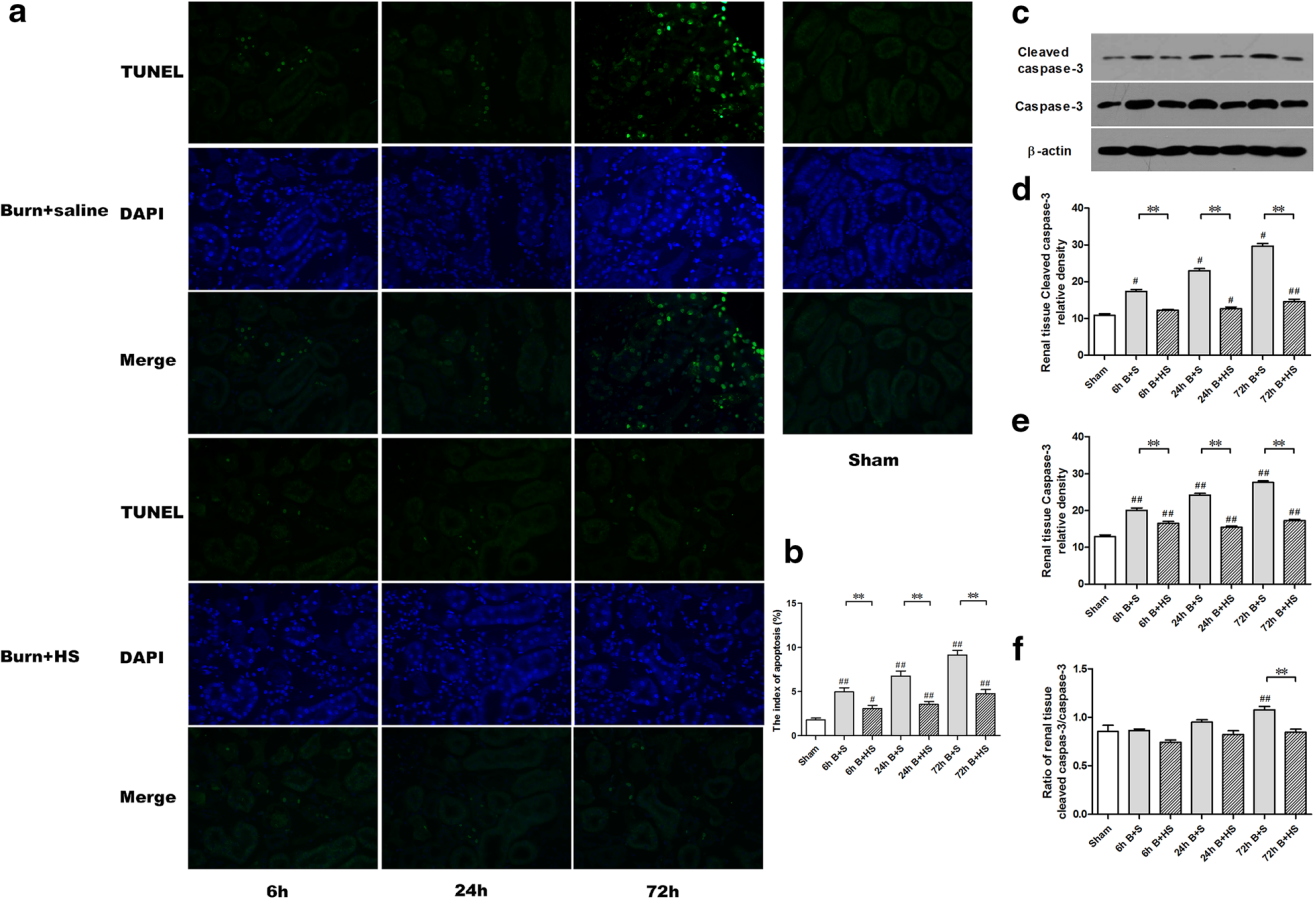
Analysis of apoptosis in renal tissues post burn. TUNEL staining revealed increased numbers of apoptotic cells at 6, 24, and 72 h post burn, whereas corresponding reductions in the time-paired groups were observed with the regular administration of HS (**a**) (vs Sham, magnification ×200). The quantitative assessment of the numbers of apoptotic cells is shown (**b**). In addition, cleaved caspase-3 protein expression, as a marker of apoptotic activation, revealed significant gradual elevations over time, and HS markedly down-regulated cleaved caspase-3 expression (**c**–**f**). The sample size was n = 5 for TUNEL staining and n = 6 for Western blotting for each group. The results were expressed as the means ± SEMs. **p < 0.01, vs Burn + saline; ^#^p < 0.05, ^##^p < 0.01, vs Sham.

As shown in Figure 4a, b, the number of tubular apoptotic cells rose significantly at 6 h post burn, gradually increased afterwards, and then continually increased to 72 h (all p < 0.05, vs Sham), whereas HS treatment substantially ameliorated burn-induced renal tissue apoptosis in the 6-, 24- and 72-h Burn + HS groups (all p < 0.05). In addition, burn injury caused a consistent elevation in cleaved caspase-3 (activated caspase-3, indicating apoptosis) levels from 6 to 72 h post burn, all of which were dramatic (all p < 0.05, vs Sham), while HS injection clearly attenuated this trend (all p < 0.05, vs corresponding Burn + saline) (Figure 4c). In terms of pro-apoptotic protein expression, the protein level of cleaved caspase-3 increased remarkably with time post burn, whilst caspase-3 showed a similar expression trend from 6 to 72 h after burn insult (all p < 0.05, vs Sham) (Figure 4d, e). After HS administration, the expressions of cleaved caspase-3 and caspase-3 at the three times exhibited drastic declines, in contrast to the corresponding Burn + saline groups (all p < 0.05) (Figure 4d, e). The ratio of cleaved caspase-3/caspase-3 represents the actual activation of caspase-3 and reflects apoptosis induction. Although the ratio of cleaved caspase-3/caspase-3 remained unchanged at 6 h post burn when compared with the Sham group, a slight increase was observed at 24 h, and a marked increase was observed at 72 h. The ratio of cleaved caspase-3/caspase-3 for the three HS treatment groups (6, 24 and 72 h) levelled out at a lower level, and a significant difference compared to the Burn + saline group occurred at 72 h post burn.

### Changes in MPO and inflammatory mediators in kidneys post burn and after HS treatment

**Figure 5:**
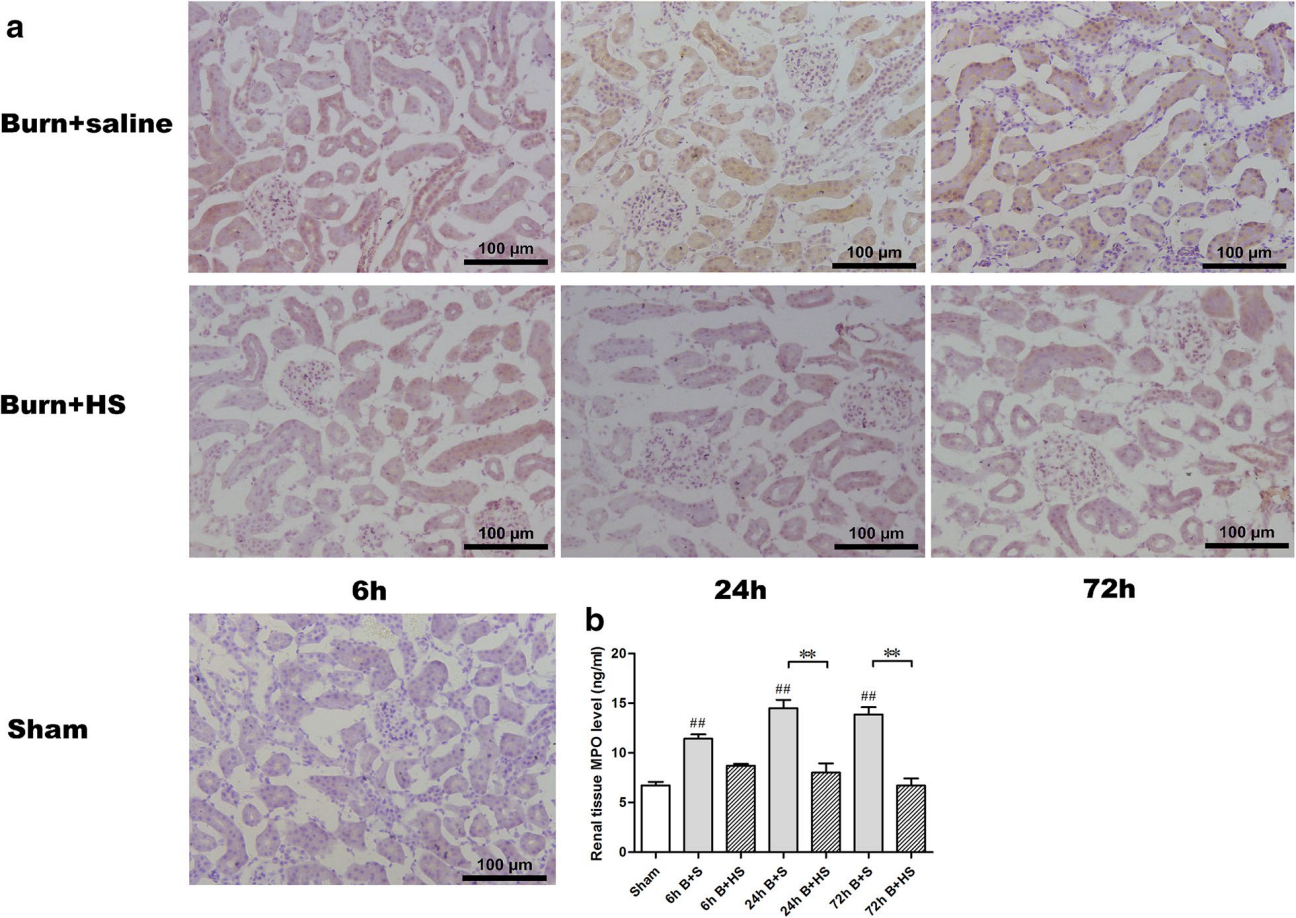
Immunohistochemistry renal tissue staining and detection of MPO protein expression in renal tissue and serum. Apparent positive staining for MPO, which is considered to be an index of neutrophil infiltration, was observed after burn insult at 6, 24, and 72 h (**a** *upper row*), whereas HS treatment decreased this phenomenon (**a** *lower row*). Based on the ELISAs, the MPO level in rat kidneys increased at all time points after burn insult, and the HS treatment ameliorated these elevated MPO levels at 24 and 72 h post burn (**b**). In addition, serum MPO levels revealed similar elevations in the MPO levels in the three Burn + saline groups; marked effects of HS application on the reversal of these elevations were also identified in the time-paired Burn + HS groups (**b**). The sample size was n = 8 for each group. The results were expressed as the means ± SEMs. **p < 0.01, vs Burn + saline; ^##^p < 0.01, vs Sham.

MPO was detected via IHC staining in rat kidneys after burn injury (Figure 5a). HS treatment was associated with decreased numbers of positively labelled cells in renal tissues (Figure 5a). Severe burn injury also led to gradually elevated renal tissue MPO levels from 6 to 72 h (all p < 0.05, vs Sham) (Figure 5b), whereas these burn-induced MPO level increases were significantly lowered in the three Burn + HS groups at 24 and 72 h (all p < 0.05) (Figure 5b).

**Figure 6:**
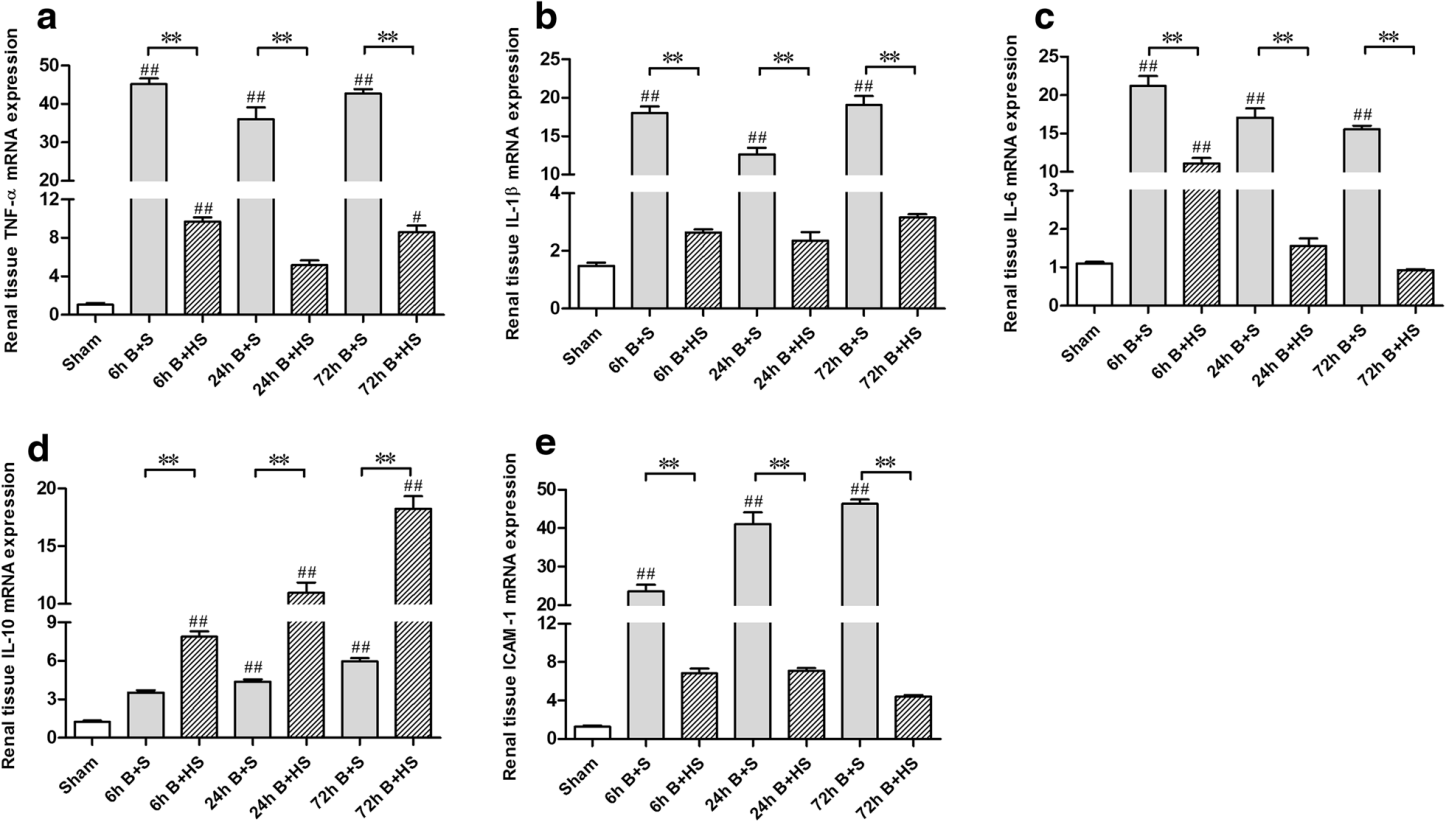
Quantitative RT-PCR analysis of inflammatory mediators in the renal tissues of severely burned rats. The qRT-PCR analysis revealed that HS treatment could significantly attenuate the clear elevation in mRNA expression after burn in the rat kidneys (**a**–**c**, **e**). Additionally, positive IHC results for IL-10 were observed post burn, and more positive renal cells were identified with HS treatment at various time points (**d**). Although the expression of IL-10 mRNA markedly increased with time, HS treatment appeared to enhance this increase in the kidneys of burned rats (**d**). The sample size was n = 6 for each group. The results are expressed as the means ± SEMs. *p < 0.05, **p < 0.01, vs Burn + saline; ^#^p < 0.05, ^##^p < 0.01, vs Sham.

Moreover, the mRNA expression of TNF-α and IL-1β in renal tissues was markedly increased at 6 h post burn (p < 0.05, vs Sham), and the extent of this increase was reduced at 24 h post burn injury but was elevated again at 72 h post burn (p < 0.05, vs Sham) (Figure 6a, b). In addition, the elevated mRNA expression of IL-6 gradually decreased over time in the rat kidneys, although all mRNA expression levels at the three time points were significantly elevated (all p < 0.05, vs Sham) (Figure 6c). The levels of both IL-10 and ICAM-1 were clearly increased at 6 h and continued to increase until 72 h post burn (all p < 0.05, vs Sham) (Figure 6d, e). After HS treatment, the mRNA expression levels of the selected inflammatory mediators were markedly reduced in all time-paired Burn + HS groups (all p < 0.05) (Figure 6a–c, e), whereas the increase of IL-10 expression became more remarkable (all p < 0.05, vs corresponding Burn + saline) (Figure 6d).

### Western blotting assessment of signalling proteins

**Figure 7:**
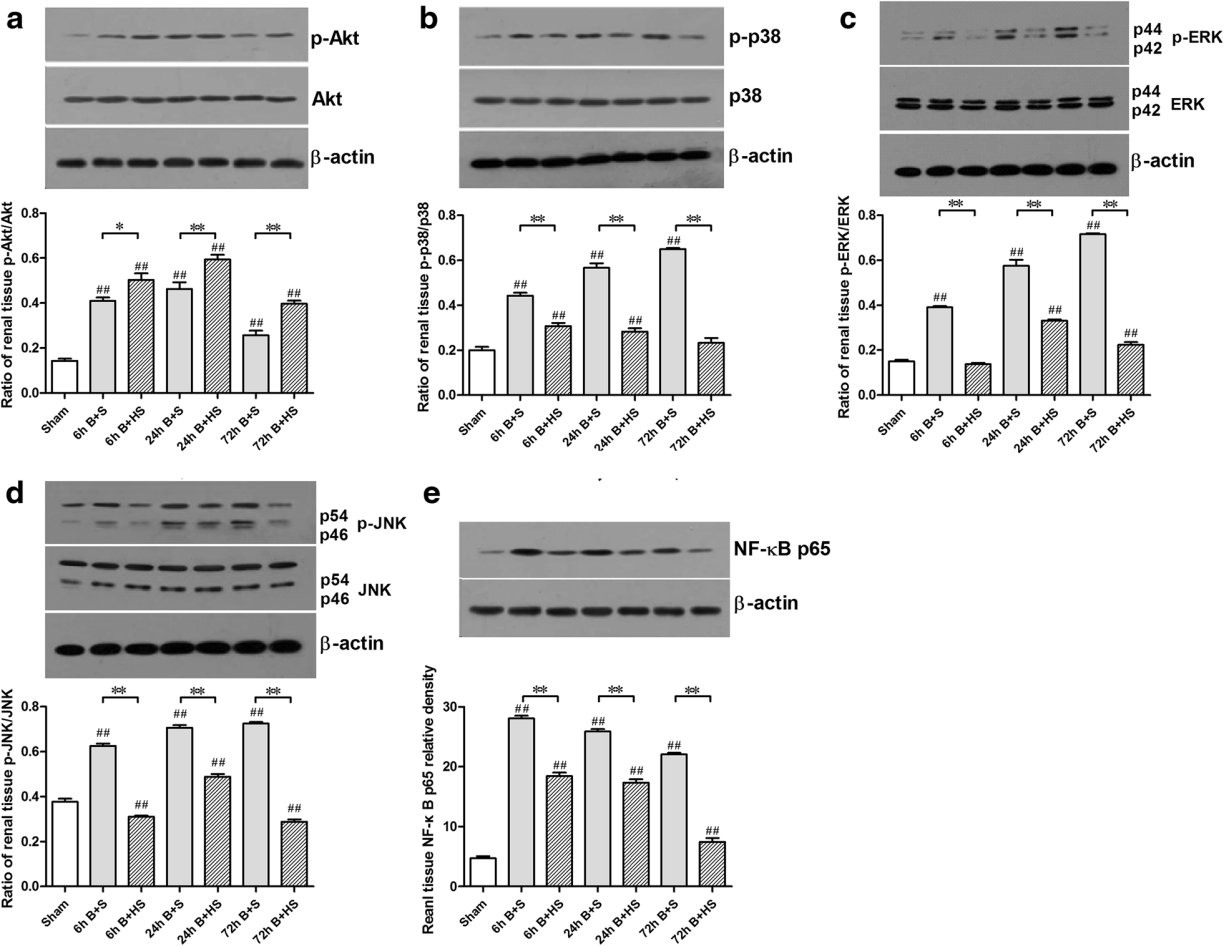
Effects of HS on signalling proteins post burn via western blotting analysis. HS relieved ROS-induced p38 phosphorylation and Akt inhibition (**a**, **b**). Both of p-JNK and p-ERK displayed marked elevations at the three time points, and HS administration decreased p-JNK and p-ERK expression (**c**, **d**). The sample size was n = 6 for each group. Following burn insult, NF-κB was markedly activated, and HS attenuated this activation (**e**). The results are expressed as the means ± SEMs. *p < 0.05, **p < 0.01, vs Burn + saline; ^#^p < 0.05, ^##^p < 0.01, vs Sham.

Following severe burn injury, phosphorylated Akt (p-Akt) levels were significantly increased at 6 h and peaked at 24 h post burn, followed by a reduction in this elevation at 72 h (all p < 0.05, vs Sham) (Figure 7a). The levels of activated p38, i.e., phosphorylated p38 (p-p38), exhibited a marked, stepwise elevation from 6 to 72 h (all p < 0.05, vs Sham) (Figure 7b). Following HS treatment, HS clearly promoted the further augmentation of the p-Akt/Akt ratio (all p < 0.05, vs corresponding Burn + saline) (Figure 7a). In contrast, the ratio of p-p38/p38 in the renal tissues of severely burned rats markedly decreased in all time groups (all p < 0.05, vs corresponding Burn + saline) and ultimately reached a similar value as that of the Sham group at 72 h (Figure 7b).

Although obvious changes in p-ERK protein expression were observed post burn (all p < 0.05, vs Sham) (Figure 7c), HS treatment led to significant down-regulation of p-ERK at all selected time points (all p < 0.05, vs corresponding Burn + saline) (Figure 7c). Furthermore, the activated pro-apoptotic kinase JNK (p-JNK) exhibited increased expression after burn injury, and the most significant elevation was observed at 72 h post burn (all p < 0.05, vs Sham) (Figure 7d); the elevation of p-JNK levels was markedly down-regulated by HS administration (all p < 0.05, vs corresponding Burn + saline) (Figure 7d).

As a subunit of the NF-κB dimer, p65 has typically been chosen as an index of NF-κB activation [29]. Although NF-κB p65 protein expression was significantly increased at 6, 24 and 72 h post burn (all p < 0.05, vs Sham), the trend towards increased expression decreased with time (Figure 7e). HS displayed an apparent effect of decreasing the increased protein expression of NF-κB p65 at all thee time points (all p < 0.05, vs corresponding Burn + saline), with the greatest reduction observed at 72 h post burn (Figure 7e).

## Discussion

**Figure 8:**
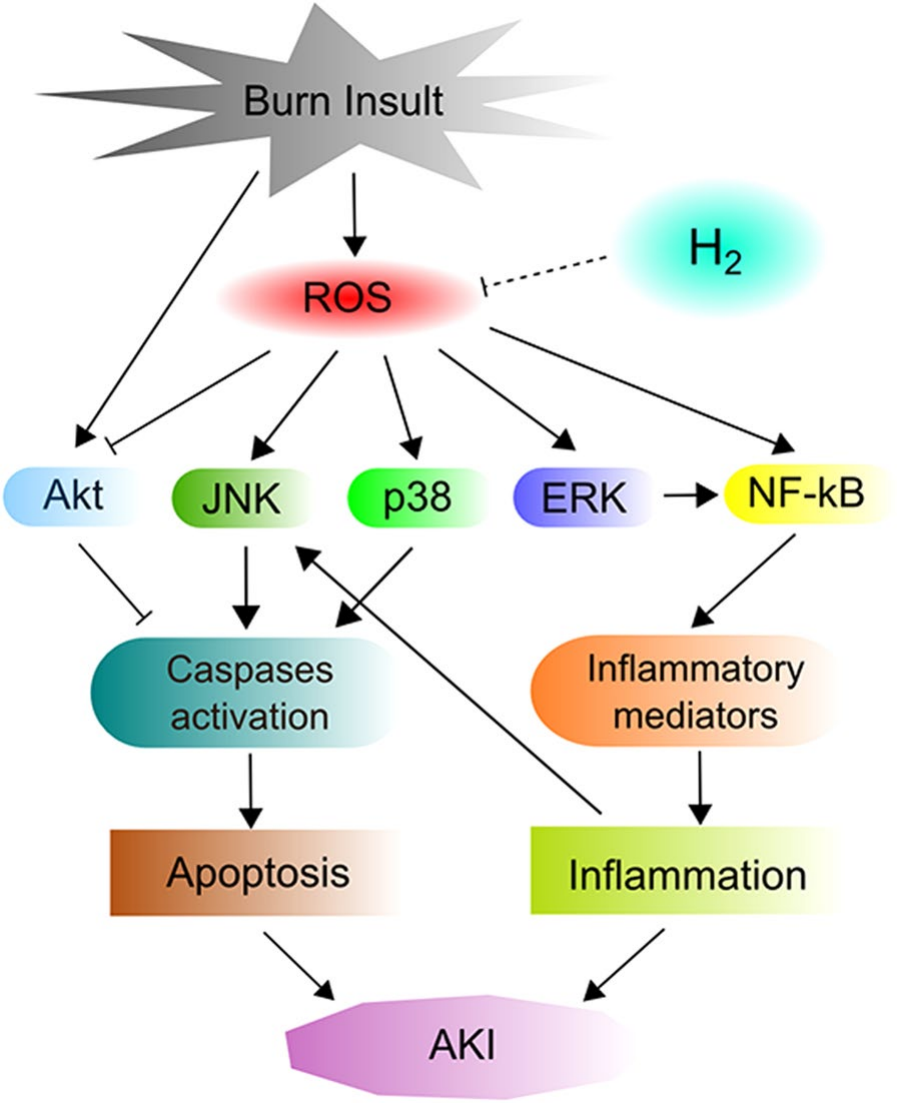
A schematic diagram of the potential mechanisms of burn-induced AKI and the routes by which H_2_ exerts its effects.

In the present study, we first explored the potential protective effect of HS on AKI after severe burn in rats. The experimental results demonstrated that HS represents a potentially novel therapeutic medium for AKI post severe burn injury that provides protection via anti-oxidative stress, anti-apoptotic and anti-inflammatory effects. Moreover, HS might further regulate the MAPKs/Akt/NF-κB signalling pathways (Figure 8).

Acute kidney injury is typically associated with injurious histological changes to renal tissue cells, including the absence of the proximal tubular brush border, blebbing of apical membranes, separation of tubular epithelial cells from the basement membrane, or aggregation of cells and proteins in the luminal region [30]. We also observed tubular epithelial necrosis by microscopic detection, similar to other studies on AKI caused by different reasons [31–33]. Applied HS, less necrotic tubules were found with a lower tubular damage score, which suggests a relief of early AKI post burn. In addition, the serum Cr and NAGL levels reflect renal function alteration and represent sensitive indicators or biomarkers of AKI [5, 30, 34–36]. Serum creatinine level elevation, including an increase of at least 0.3 mg/dl or 50%, combined with a reduction in urine output (documented oliguria of less than 0.5 ml/kg per hour for more than 6 h), is also one of the current standard criteria for AKI diagnosis [37]. After the critical burn insult, the rat kidneys presented progressive histologic injuries with time, in parallel renal dysfunction was also observed according to the serum Cr and NAGL results. The regular management of HS via the IP route effectively ameliorated renal tissue damage and improved burn-induced renal dysfunction, all of which suggested the protective potential of HS on early AKI post burn.

Substantial evidence has suggested that ROS and oxidative stress play vital roles in the pathogenesis of AKI, and similar effects have been verified in other burn-induced tissue injuries [38–42]. Because of the abundance of polyunsaturated fatty acids, the kidney is considered vulnerable to ROS-mediated oxidative stress, with the ability to generate ROS itself [43, 44]. Moreover, the aberrant and excessive production of free radicals such as ·OH and ONOO^−^ results in vascular constriction and immoderate consumption of endogenous anti-oxidant enzymes such as SOD, GSH-Px, and CAT, eventually giving rise to organ injury [13, 23, 45]. In our study, HS exhibited a strong ability to attenuate severe burn-induced oxidative stress damage to renal tissues by scavenging free radicals and up-regulating endogenous anti-oxidant enzyme activities.

Apoptosis is a common response of the kidney when confronting insults such as a burn, ischaemia, radiation, trauma, or toxic injury [46–49]. Mariano et al. demonstrated that pro-apoptotic mediators in the circulatory system contribute to renal functional alterations after burn injury [47]. Furthermore, ROS-related oxidative stress induces mitochondrial dysfunction and tubular epithelial cell apoptosis after burn injury via triggering a series of apoptotic mediators [30, 50]. We observed the obvious induction of apoptosis in renal tissue post burn, and persistent ROS-related oxidative stress played roles in the progression of apoptosis. Similar to prior reports, our results of increased cleaved caspase-3 expression and number of TUNEL-positive cells, which corresponded to the prior renal function evaluation results, indicated that the most severe AKI conditions occurred at 72 h, following increased renal failure. In this study, HS treatment significantly reduced renal cell apoptosis in the rat burn model, and this was verified to be an anti-apoptotic effect.

The inflammatory response also participates in the onset and development of early AKI post burn [51, 52]. Generally, cell necrosis can cause the loss of cell membrane integrity and the uncontrolled release of products of cell death into the extracellular space, all of which can initiate an inflammatory response in the surrounding tissue and finally result in advanced tissue injury [53]. Therefore, as well as systemic response caused by local burn wound, the tubular necrosis also contributes to the inflammatory injury in kidneys of burned rats [54, 55]. The extent of inflammation can indirectly reflect the level of tissue necrosis. In addition, the release of circulatory and tissue inflammatory mediators might cause increased vascular permeability and tubular damage, which could eventually lead to filtration failure and tubular dysfunction. In terms of inflammatory mediators, ROS-related oxidative stress may initiate inflammatory cascades that result in pathophysiological organ changes [56]. Inflammatory cytokines and adhesion molecules such as TNF-α, IL-1β, IL-6, and ICAM-1 trigger a strong inflammatory response to injurious stimuli [4], whereas MPO signifies an inflammatory condition by reflecting the infiltration of neutrophils [40]. IL-10 is an anti-inflammatory cytokine that is generated by monocytes/macrophages and T and B lymphocytes, which antagonise the inflammatory response and regulate auto-immunity [57]. We observed and examined renal tissue MPO levels using IHC staining and ELISA and discovered that the MPO levels of rat renal tissues were markedly augmented post burn, indicating the potentially important role of inflammatory cell infiltration in tissue injury post burn. The study conducted by previous researchers demonstrated that H_2_ could effectively attenuate LPS/burn-induced lung neutrophil recruitment and inflammation supported by the down-regulation of tissue MPO and proinflammatory cytokines (TNF-α, IL-1β, IL-6) [20, 40]. In addition, Cardinal et al. suggested that administration of hydrogen water (similar to HS) significantly attenuated the intragraft production of inflammatory cytokines (TNF-α, IL-6, ICAM-1 and INF-γ) after kidney allotransplantation [21]. In several in vivo and in vitro studies, H_2_ was reported to bring about a further increase in IL-10 levels after endotoxin stimulation as a way to attenuate inflammation [58, 59]. In our study, the final results showed that H_2_ ameliorates AKI after severe burn in rats through an anti-inflammatory effect that involves the down-regulation of inflammation-related enzymes and cytokines and up-regulation of anti-inflammatory cytokines in the circulation and local tissues.

Afterwards, we estimated the potential value of MAPKs and Akt signalling in the beneficial effects of H_2_ on early AKI post burn. As a family of serine/threonine protein kinases, MAPKs are considered to be responsible for most cellular responses to cytokines and external stress signals and are vital for the induction of apoptosis and inflammatory mediators [60, 61]. In terms of p38 MAPK, its activation has been reported to participate in the induction of apoptosis and inflammatory response in local wounds and remote organs after burn insults [54, 62–66]. In addition, previous studies also indicated that p38 MAPK plays an important role in the development of renal injuries via its regulation on renal cell apoptosis and the release of local or systemic inflammatory mediators [67–69]. Feng et al. demonstrated that phosphorylated p38 MAPK, regarding as an activated condition, plays a more important role in the induction of renal cell apoptosis, which contributes to the renal injury post burn [30]. In the present study, we observed a similar activation of p38 MAPK in the renal tissue after burn, paralleling with the enhanced tubular apoptosis and the release of pro-inflammatory cytokines, which suggests p38 MAPK may be involved in the progress of early AKI post burn through regulating burn-induced apoptosis and inflammation. In common knowledge, JNK has been implicated in mitochondrial death and its activation contributes to cell apoptosis [11, 70]. Several previous studies reported that JNK activation in renal samples was associated with tubular cell apoptosis after renal ischemia–reperfusion [13]. Recently, Marshall et al. demonstrated the silence of JNK2 could alleviate the hepatic apoptosis post burn [71]. In addition, it was reported that the JNK pathway plays a critical role in the smoke-induced lung injury and the application of JNK inhibitor could attenuate the airway apoptosis [72]. Except ROS, JNK can also be activated by some pro-inflammatory cytokines including TNF and IL-1 [73]. Due to a significant increase of JNK phosphorylation and corresponding tubular apoptosis in rats’ kidney post burn, we speculated it may play an important role in regulating burn-induced tubular apoptosis. ERK, another important member of MAPKs, has been suggested that its activation is associating with cell survival in several kinds of renal injuries. However, in this study, the burn-induced activation of ERK in rats’ kidney did not company with the corresponding decrease of tubular apoptosis. Therefore, ERK may be involved in the other aspect of the mechanism of burn-induced AKI rather than inhibiting tubular apoptosis. In an in vivo study, Seo and collaborators demonstrated that activated ERK induces the dissociation of IκBα from NF-κB, therefore allowing nuclear translocation and DNA-binding of NF-κB and the subsequent production of pro-inflammatory cytokines [74]. The result of our study exerted that the phosphorylation of ERK, as activated ERK, was paralleled with the increase of NF-κB p65 expression and the release of pro-inflammatory mediators, which suggests ERK participates in the regulation of tissue inflammation during the process of burn-induced early AKI. In view that ROS can trigger activation of signaling pathways involved in cell migration and invasion such as MAPKs, H_2_ is like to influence some members of ROS to achieve its effect [75]. Although H_2_ has been observed to influence some signal transduction pathways, some researchers believe that this is based on its role as an indirect modulator rather than as a molecule binding directly to signalling receptors. In contrast, Itoh and colleagues regarded H_2_ as a gaseous signalling molecule due to the result showing that H_2_ could attenuate the phosphorylation of FcεRI-associated Lyn as well as its downstream signal transduction (such as JNK, p38 MAPK, ERK) in rat RBL-2H3 mast cells, followed by the inhibition of NADPH oxidase activity and reduction of hydrogen peroxide (H_2_O_2_) levels [76]. In addition to ROS activation, JNK can be activated by the LPS-induced inflammatory response and attenuated by hydrogen inhalation [77]. The data, obtained from human histocytic lymphoma U937 cells, indicated that H_2_O_2_ and ·OH could activate PI3K-Akt and PLC-Ras-Raf-ERK signaling pathways [78], whereas Xu and Zhang discovered that saturated HS decreased LPS-induced ERK phosphorylation in a rat model of acute liver dysfunction [79]. Moreover, Sobue et al. indicated that H_2_ can attenuate ERK, p38 MAPK, and NF-κB activation in mouse livers [80]. Taken together, we suggested H_2_ may inhibit NF-κB activation by reducing oxidative radicals-induced ERK phosphorylation, which allows degradation of IκBα from NF-κB. Correspondingly, Akt is an important downstream signal of the classic PI3K-Akt pathway, and Akt activation has been regarded as a cell survival factor that antagonises apoptosis in renal tissue exposed to heavy metal or burn insult [30, 61]. The protective effects of Akt in apoptosis include enhancing the capacities of antioxidant and anti-apoptotic proteins and reducing the capacities of pro-apoptotic proteins [61]. Moreover, the significant activation of Akt in rat kidneys was observed in the early stage after burn insults, while a late decrease in Akt activation appeared with increased ROS [30, 81]. Considering the other possible routes resulting in the activation of Akt and our results, the effect of H_2_ on Akt phosphorylation may be attributed to its effect on ROS-mediated Akt inhibition. Taken together, we determined that the regular administration of HS could down-regulate the p-p38/p38, p-JNK/JNK, and p-ERK/ERK ratios, as well as up-regulate Akt phosphorylation.

In a series of previous experiments, NF-κB signalling was implicated in AKI induced by various stimuli, such as I/R injury, haemorrhagic shock, lipopolysaccharide (LPS), or cisplatin, via interactions with TNF-α, IL-1β, ICAM-1, etc. [82–88]. In addition, ROS has been reported to participate in NF-κB pathway activation, which is unsurprising given the oxidant-sensitive properties of NF-κB [86]. The protective effect of H_2_ has been manifested in several models of inflammatory injury based on its inhibition of inflammatory cell infiltration, NF-κB activation and pro-inflammatory cytokine production [23]. Furthermore, H_2_ has been reported to inhibit cytokine-induced LOX-1 gene expression by directly suppressing IκBα to down-regulate NF-κB activation [89]. On the other hand, the increase in infection-caused free radicals contributed to the subsequent degradation of cytosolic IκBα and to the nuclear translocation of NF-κB subunits (p65 and p50), and H_2_ indirectly inhibited NF-κB signalling through reducing oxygen-free radicals [13]. ERK may be also involved in regulation of H_2_ on NF-κB signalling, considering its reported regulation on NF-κB activation [74]. Our results indicate that HS may protect the kidneys of burned rats from AKI via negatively regulating NF-κB signalling.

## Conclusion

In summary, the present study first demonstrates the protective effects of H_2_ against early AKI following severe burn in rats. The beneficial effects of this treatment are a result of its ability to relieve oxidative stress, apoptosis and inflammation and may be mediated by the complex modulation of the MAPKs, Akt and NF-κB signalling pathways.
